# Mutations in p53 Gene Exons in a Sample from the South of Spain in Oral Cancer

**DOI:** 10.4317/jced.58799

**Published:** 2021-10-01

**Authors:** Guiomar Martín-Lozano, Raquel Gómez-Díaz, Fernando Iglesías-Martín, Daniel Torres-Lagares, Aida Gutiérrez-Corrales, José-Luis Gutiérrez-Pérez

**Affiliations:** 1 Oral and Maxillofacial Surgery Unit, Virgen del Rocío University Hospital, Seville, Spain; 2 Instituto de Biomedicina de Sevilla, Seville, Spain; 3 Oral and Maxillofacial Surgery Unit, Virgen del Rocío University Hospital, Seville, Spain; 4 Full Professor of Oral Surgery at Dental School. University of Sevilla, Seville, Spain; 5 Oral Surgery Department, Dentistry Faculty, University of Seville, Seville, Spain; 6 Oral and Maxillofacial Unit, Virgen del Rocio Hospital, Seville, Spain. Oral Surgery Department, Dentistry Faculty, University of Seville, Seville, Spain

## Abstract

**Background:**

Cancer is a genetic disease caused by mutations in DNA and epigenetic alterations that control gene expression. The majority of epidermoid carcinomas develop within the fields of epithelial genetic alterations. The mechanisms underlying tumorigenesis of epidermoid carcinoma are as yet unknown; therefore, precise identification of the risk factors is needed. Aim: The main aim of this study is to analyse and identify the emergence of the mutations described in the literature of the p53 gene with regard to the emergence of cancer in a sample of dysplastic and cancerous lesions in oral cavity mucosa in the population of the south of Spain, in order to determine the presence of said mutations and the percentage of them in our population.

**Material and Methods:**

A cross-sectional study was carried out, with a sample size of 22 patients with potentially malignant oral lesions ancillary to biopsy. All were patients, of both sexes, over 18 years of age from the Virgen del Rocío University Hospital with potentially malignant lesions in oral mucosa ancillary to biopsy (leukoplakias, erythroplasias or leukoerythopkias). An anatomopathological study was performed on all the samples and the lesions were divided into three types: low-grade dysplasia, high-grade dysplasia and squamous cell carcinoma. In respect of the genome study process, a complete search or scan for mutations in exons 5, 6, 8 and 9 of the p 53 gene was carried out, given that in the IARC database we observed that the 5 and 6 as well as the 8 and 9 exon sizes can be scanned completely in this way, since they have amplificon sizes of 476 and 445 base pairs respectively.

**Results:**

In the scan for the complete exons 5, 6, 8 and 9 only a single result of interest was found to be described. In patient NBI 57 a change was observed in the TAT triplet by ATT of EXON 6, the change being of the T nucleotide by the A and in both directions both in Forward and Reverse. The exact location in the NCBI is GR Ch 37 p13 on chromosome 17, EXON 6 of the P53 gene and the change is in the C.613 T>A nucleotide; NM_000546.

**Conclusions:**

On reviewing this genetic variant in different scientific databases, such as ENSEMBL among others, in at least 6 different biocomputing tools it is described as a pathogen, therefore we can conclude that it is a pathogenic mutation for this case in particular. The rest of the mutations described in the literature on exons 5, 6, 8 and 9 of the p53 gene have not been found in our sample.

** Key words:**Oral cancer, p53, Mutations, Exon.

## Introduction

Oropharyngeal cancers are amongst the ten most frequent neoplastic tumours in the world, with an estimated incidence of approximately 275,000 cases of isolated oral cancer per year (1,2). Oral cancer is the fifth most common cancer in the world (3). More than 90% of oral cancers affect the lips, gum, tongue, buccal mucosa, floor of the mouth and the hard palate, arising from the squamous epithelium hence being termed oral squamous cell carcinomas, or epidermoid carcinomas. After developing an initial carcinoma, there is a high risk of developing a second primary malignant tumour in the aero-digestive mucosa (4).

Patients with oral cancer are typically men aged over 40 with a history of frequent exposure to risk factors such as smoking, alcohol, malnutrition, lack of oral hygiene, etc.; however, there is a cohort of younger patients with lower accumulated exposure to alcohol and smoking who, with increasing frequency, are presenting with oropharyngeal cancer. These latter cases are associated with the human papillomavirus (2,4).

Despite major progress in the treatment of oral cancer in the last 30 years, the 5-year survival rate remains around 50% and can drop to 15% in cases which present as advanced stage cancers with cervical metastases (5). These Figures are mainly due to a late diagnosis in general, with approximately half of oral cancers being diagnosed in stages III or IV (6). Consequently, early diagnosis and treatment of tumours is essential to improve survival rates. Early detection of oral cancer consists in the diagnosis of premalignant lesions such as in situ dysplasia or carcinoma. This early diagnosis improves morbidity and mortality (7,8). It must be borne in mind that treatment in advanced cases is usually disFiguring, painful and invasive, often resulting in loss of function and reduction in the quality of life. However, it is difficult to detect oral cancer in the early stages, due to it being relatively asymptomatic and presenting as subtle changes in the epithelium which makes it difficult to see using white light inspection (2).

These changes in mucosa may appear as white, red or combined staining, and are called leukoplakias, erythroplasias or erythroleukoplakias respectively. There are other less frequent lesions, but also potentially malignant such as oral lichen planus and lichenoid lesions, chronic candidal hypertrophy and proliferative verrucous leukoplakia (9). All in all, these lesions are the ones that are known as potentially malignant (8) and have a prevalence of 2.5% in the population (4).

Although only a small proportion of potentially malignant lesions will ultimately become malignant, it is believed that the key to improving a patient’s prognosis is through early detection and management of these lesions.

To achieve better survival results in the handling of squamous cell oral carcinoma intervention needs to take place in the initial stages. Early detection and diagnosis of patients with risk factors and potentially malignant oral lesions must be undertaken. A selection must be made among patients with dysplastic lesions in the environment of multi-disciplinary primary care and specialist care (10).

Cancer is a genetic disease caused by mutations in DNA and epigenetic alterations that control gene expression. The majority of epidermoid carcinomas develop within the fields of epithelial genetic alterations. The mechanisms underlying tumorigenesis of epidermoid carcinoma are as yet unknown; therefore, precise identification of the risk factors is needed (11).

Currently genetic markers are being sought to evaluate the risk of malignant transformation (12). The persistence of genetic changes after treatment poses an important challenge, because it can lead to a recurrence or the emergence of further tumours which are a consequence of a large percentage of deaths (13). According to several authors (14-16), the presence of mutations in TP53 plays a fundamental role in the formation of pre-cancerous fields and propose that the mutations of p53 in the epithelium of head and neck is the first manifestation of carcinogenesis in said territory.

Mutations of p53 have also been observed in a variety of dysplastic epithelial lesions in several tissues, including the oral cavity. This leads us to think that mutations of the p53 gene may be an early event in the squamous epithelium during the progression to cancer (17).

The p53 gene is located in the short arm of chromosome 17p13.1 which contains 11 exons and is a suppressor gene in carcinogenesis because under normal conditions it acts as a negative regulator of cell proliferation increasing the amount of p53 nuclear protein; interrupting the cell cycle by inducing p21 protein which blocks the replication of DNA and ultimately inducing cell apoptosis (18).

Mutation of the p53 tumour suppressor gene is one of the most frequent genome changes in human cancers. Immunohistochemical demonstration of p53 protein cannot be assumed to be an absolute marker for p53 gene mutations, besides its expression is seen in half of oral cavity carcinomas at advanced stages (19). More than 90% of the mutations described for this gene are located on exons 8-5 (20).

The presence of mutations in TP53 seems to be an interesting biomarker for the screening of potentially malignant oral mucosa and apparently predicts malignant progression (21,22).

Furthermore, some TP53 mutations may be easily detected by immunohistochemistry of p53 protein, such that immunohistochemical testing could be a really simple alternative to mutation testing.

Conversely, mutations of p53 gene increases the risk of the emergence of other synchronic neoplasias. Given that, at least that we know of, the detailed study of the mutations of the P53 gene in squamous cell cancer and in potentially malignant lesions has not be undertaken up to now in Spain, this study was carried out to identify the mutation in different exons in the gene in patients with the previously described lesions.

Consequently, the main aim of this study is to analyse and identify the emergence of the mutations described in the literature of the p53 gene with regard to the emergence of cancer in a sample of dysplastic and carcinomatous lesions of oral cavity mucosa in the south of Spain population, with a view to determining the presence of said mutations and the percentage of these in our population.

## Material and Methods

Retrospective case study and controls and approved by the corresponding Ethics Committee (Hospital Virgen del Rocío Ethical Committee – Exp PI-0081-2016). The study is descriptive and observational where the only invasive procedure on patients is the collection of a small amount of blood and a dental examination. The patient (or the person responsible for them) gives their consent based on the direct benefits for the research patient.

A cross-sectional study was carried out, with a sample size of 22 patients with potentially malignant oral lesions ancillary to biopsy.

The study population is made up of patients of both sexes from “Virgen del Rocío” University Hospital, Seville, who attended the Oral and Maxillofacial Surgery Unit for the assessment of potentially malignant oral lesions. Patients met each and every one of the inclusion criteria and none of the exclusion criteria. Surgery was performed over a period of 18 months. All patients were likewise subjected to an operation protocol. They were duly informed about the nature of the study and gave their express consent.

Among the pre-inclusion criteria were: patients of both sexes over 18 years of age who attend the Virgen del Rocío University Hospital with potentially malignant oral lesions in oral mucosa ancillary to biopsy (leukoplasias, erythroplakias or leukoerythroplakias) and agreed to take part in the study by signing an informed consent form. As an inclusion criterion and once biopsies had been taken of the lesions, the results were found to be dysplasias or carcinomas.

Among the exclusion criteria were patients with vascular malformations, general poor state of health preventing examination of the patient and sampling, those under-age and patients with intellectual disability unable to understand and sign the informed consent form.

Once the inclusion and exclusion criteria had been met the operation protocol and information gathering was carried out. A conventional oral examination was performed based on the inspection and palpation of the lesion. If the clinician considered that a biopsy of the lesion could be taken, the patient was invited to take part in the study. The clinician would record the biopsy site deemed most suiTable.

Left (reverse) exon 5-6 P53 / 20mer / 0,0250 UMO / Desalted Purification

TGTTCACTTGTGCCCTGACT(5`-3`) SIGM A Aldrich (St Louis, MI, USA).

Right (forward) exon 5-6 P53 / 20mer / 0,0250 UMO / Desalted Purification

TTAACCCCTCCTCCCAGAGA (5`-3`) SIGM A Aldrich (St Louis, MI, USA).

## Results

Patient characteristics: Some 22 patients were recruited between November 2017 and May 2019 (18 months) diagnosed with lesions in oral mucosa a priori potentially malignant, 9 women and 13 men, with ages grouped in under 60 years, 10 patients and over 60 years, 12. We divided patient lesions based on location in the oral cavity including those appearing in retromolar trigone, 1; buccal mucosa, 4; ventrolateral tongue, 7; labial mucosa ,1; floor of the mouth, 3; dorsal tongue, 2; attached gingival,1; and palate, 3 patients.

Concerning patient habits we computed alcohol consumption (non-drinkers, 17, drinkers, 4, and ex-drinkers, 1 patient); smoking (non-smokers 8, ex-smokers 4, smoking less than a packet a day 6, and smoking more than a packet a day, 4 patients) oral hygiene and brushing of teeth (three times a day, 5, once a day, 15 and less than seven times a week, 2).

Aspects of patients such as history of prior oral carcinoma were analysed (19 had none and 3 patients had), having received radiotherapy (no patients had received it) and systemic diseases (19 healthy patients, and 3 with a systemic disease).

Recorded characteristics of oral mucosa lesions were type of lesion (leukoplasia, 11 patients, erythroplakia, 4, leukoerythroplakia, 5 and ulcerative lesions, 2); size of lesion (less than 1 cm, 5 patients, between 1 and 3 cm, 14 and greater than 3 cm, 3 patients); if there had been underlying induration or not (in 5 patients it had existed); and whether spontaneous pain had occurred (no pain, 17 patients and pain, 5); days of progression grouped in less than 850 days, with 18 patients, and more than 850 days, 4; and finally the pathological anatomy results were classified according to (low-grade dysplasia, 15, high-grade dysplasia, 2, and carcinoma, 5 patients).

In respect of the gene study, no patient in our population in the south of Spain was found with point mutation described in exon 5 of the P53 gene as a transition from T base to C base in codon 270 (c.T 270 C).

This made us consider continuing to search for the other point mutations described in the reference article (14) in exons 5, 6, 7 and 8. This exploration did not provide the same result of absence in our population. Bearing in mind that the size of the P53 gene exons is no higher than 500 base pairs, it was more appropriate to review the complete exons. That way we would be more rigorous given such a small cohort of patients and the low probability of finding the point changes described.

The analysis was performed on sequences in both directions to dismiss false positives, as has been described in the Materials and Methods section, employing Codon Code Alignery software (Centerville, MA, USA).

In the scanning of complete exons 5, 6, 8 and 9 just one interesting result worth describing was found. In patient NBI 57 a change was observed in the TAT triplet by ATT of EXON 6, the change being of the T nucleotide by the A and in both directions both in Forward and Reverse. In this patient, the pathological anatomy report was of low-grade dysplasia. It was a man whose lesion was located in the floor of the mouth, a drinker, 46 years of age, smoking less than one packet of cigarettes a day. The lesion had a leukoplastic appearance, less than one centimetre in size, with a 60-day course and not indurate. He had no history of carcinoma or prior radiotherapy, but he did present systemic diseases. The exact location in the NCBI is GR Ch 37 p13 on chromosome 17, EXON

6 of the P53 gene and the change is in the C.613 T>A nucleotide; NM_000546. On reviewing this genetic variant in different scientific databases, such as ENSEMBL among others, in at least 6 different biocomputing tools it is described as a pathogen, therefore we can conclude that it is a pathogenic mutation for this case in particular.

## Discussion

In the genetic reference article (14) point mutations for P53 gene are described, a transition in codon 270 of exon 5 and a deletion in the codon 288 on exon 8. None of them have been found in any case in our patient cohort, in the south of Spain population. It may be that there are different mutations in our sample since in the various existing publications there is no consensus for correlating them (17). Neither can we relate the mutations of p53 in respect of other populations, since, even by studying them using different techniques, they have not been determined (20).

Several cytogenetic studies have described genetic abnormalities in oral epidermoid carcinoma, (21) such as for example specific changes in chromosome segments in respect of loss or gain in these (22) but we have found no descriptions of mutations in potentially malignant oral cavity lesions in the Spanish population of the south of Spain like ours.

The patient sample in our study is limited, there are only 22 patients, but they represent the whole spectrum of malignant and potentially malignant lesions in oral mucosa, indicating in the case of the potentially malignant ones the degree of pathologic dysplasia.

To ensure in such a small cohort that we do not dismiss any other interesting change, we considered whether it would be possible, instead of searching for point mutations, to review the sequences of complete exons, in this way we would have more chances of finding a mutation in our population.

We observed that the size of exons 5 and 6 are 476 base pairs and in the case of the 8 and 9 exons there are 445 base pairs. Both, therefore, are easy to tackle by means of adequate oligonucleotide design, amplification with PCR and sequencing analysis as we have described earlier.

## Conclusions

After carrying out the gene study we can conclude that, in our cohort of patients in the south of Spain, one case of low-grade dysplasia was found in the pathology anatomy report which presented genetic change in exon 6, from a T to an A, in nucleotide 613 and identified with the coordinate value rs 1057520008.

Hence, we also conclude that, on reviewing this genetic variant in different scientific databases, such as ENSEMBL among others, in at least six different biocomputing tools it is described as pathogenic, consequently, we can conclude that it is a pathogenic mutation for this specific case.

The rest of the mutations described in the literature on exons 5, 6, 8 and 9 of the p53 gene have not been found in our sample.
